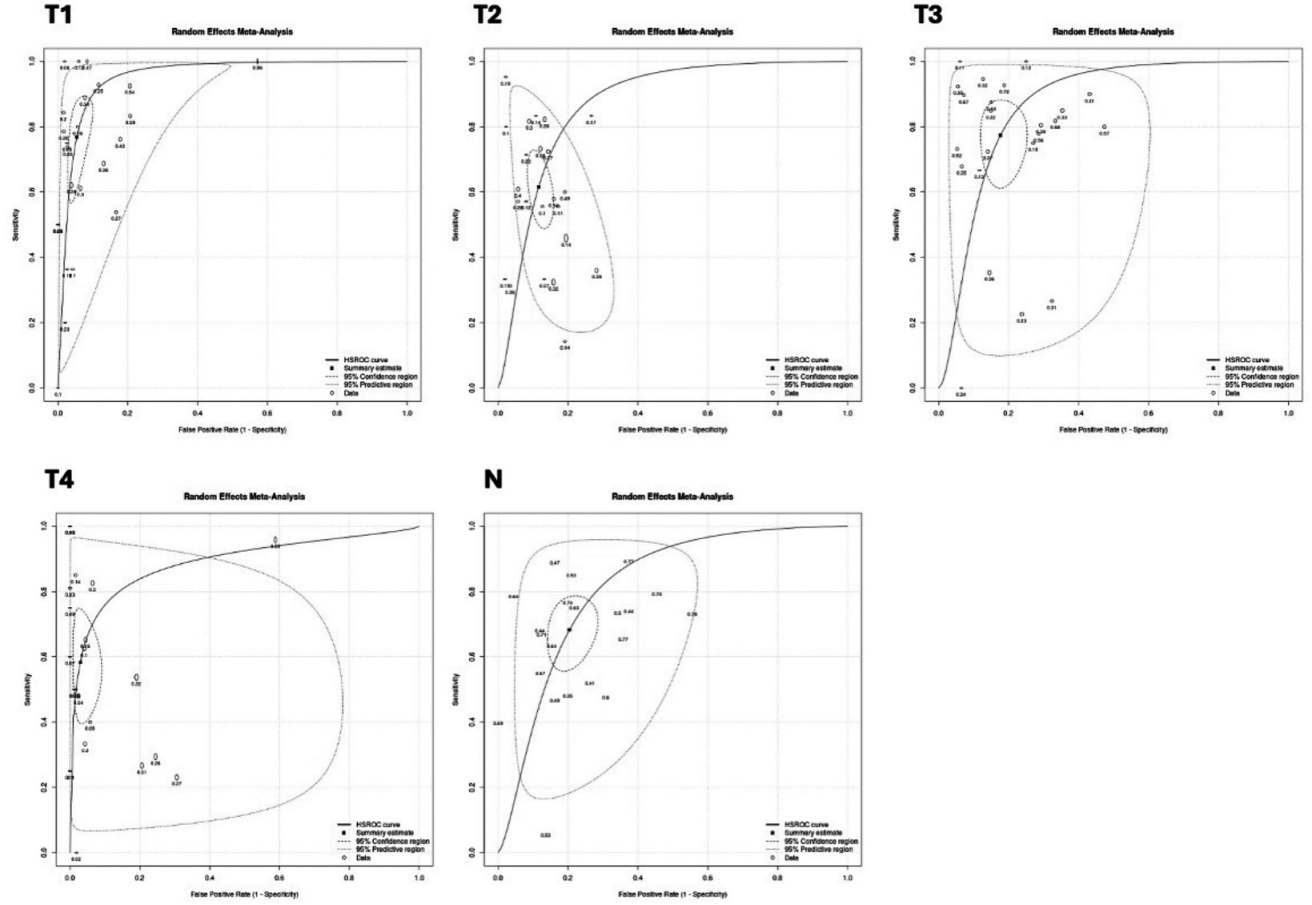# Poster Session I - A158 DIAGNOSTIC PERFORMANCE OF ENDOSCOPIC ULTRASOUND FOR GASTRIC CANCER STAGING: A SYSTEMATIC REVIEW AND META-ANALYSIS

**DOI:** 10.1093/jcag/gwaf042.158

**Published:** 2026-02-13

**Authors:** K Zheng, K Khalaf, T Rizkala, Y Alasmi, M A Bucheeri, M Harb, M Hu, R Jayawardena, H Li, C Na, T Nishimura, A Slotin, M Youssef, Y Yuan, G May, N Calo, J Mosko

**Affiliations:** Medicine, Div of Gastroenterology, St Michael’s Hospital, Toronto, ON, Canada; Medicine, Div of Gastroenterology, St Michael’s Hospital, Toronto, ON, Canada; Humanitas University, Milan, Italy; Medicine, Div of Gastroenterology, St Michael’s Hospital, Toronto, ON, Canada; Medicine, Div of Gastroenterology, St Michael’s Hospital, Toronto, ON, Canada; Medicine, Div of Gastroenterology, St Michael’s Hospital, Toronto, ON, Canada; Medicine, Div of Gastroenterology, St Michael’s Hospital, Toronto, ON, Canada; Medicine, Div of Gastroenterology, St Michael’s Hospital, Toronto, ON, Canada; Medicine, Div of Gastroenterology, St Michael’s Hospital, Toronto, ON, Canada; Medicine, Div of Gastroenterology, St Michael’s Hospital, Toronto, ON, Canada; Medicine, Div of Gastroenterology, St Michael’s Hospital, Toronto, ON, Canada; Medicine, Div of Gastroenterology, St Michael’s Hospital, Toronto, ON, Canada; Medicine, Div of Gastroenterology, St Michael’s Hospital, Toronto, ON, Canada; McMaster University, Hamilton, ON, Canada; Medicine, Div of Gastroenterology, St Michael’s Hospital, Toronto, ON, Canada; Medicine, Div of Gastroenterology, St Michael’s Hospital, Toronto, ON, Canada; Medicine, Div of Gastroenterology, St Michael’s Hospital, Toronto, ON, Canada

## Abstract

**Background:**

Accurate loco-regional staging is essential in the management of gastric cancer, guiding decisions regarding neoadjuvant therapy, surgical and endoscopic interventions. Endoscopic ultrasound (EUS) is widely used for evaluating tumor depth (T stage) and regional lymph node involvement (N stage), but reported diagnostic performance varies across studies.

**Aims:**

This systematic review and meta-analysis aimed to synthesize evidence on the accuracy of EUS for staging gastric cancer.

**Methods:**

A comprehensive literature search was performed in MEDLINE, Embase and Cochrane Library until May 27, 2025 to identify studies reporting the diagnostic performance of EUS for gastric cancer staging compared with histopathology as the reference standard. Pooled sensitivity, specificity, and accuracy for T and N staging were calculated using a bivariate random-effects model. Heterogeneity was assessed with the I2 statistic, and publication bias was evaluated using Deeks’ funnel plot asymmetry test.

**Results:**

Across 33 studies including 4,444 patients, conventional EUS demonstrated high diagnostic performance for T staging of gastric cancer. Accuracy was high, ranging from 80% (95% 0.75–0.83) for T3 lesions to 90% (95% CI 0.87–0.93) for T1 lesions. Heterogeneity was substantial (I2 = 82.0–93.7%), reflecting variability in study design, patient populations, and operator expertise. Overstaging and understaging occurred in 14% (95% CI: 0.09–0.20) and 11% (95% CI: 0.09–0.13) of lesions, respectively. Pooled sensitivity ranged from 58% (0.46–0.70) for T4 lesions to 76% (95% CI 0.64–0.87) for T1 lesions. Pooled specificity ranged from 82% (95% CI 0.77–0.86) for T3 lesions to 95% (95% CI 0.93–0.97) for T1 lesions. Area under the curve (AUC) ranged from 0.838 for T4 lesions to 0.924 for T1 lesions. For nodal staging, data from 22 studies encompassing 2,036 patients demonstrated more modest performance. Pooled sensitivity was 67% (95% CI: 0.59–0.75) and specificity was 79% (95% CI: 0.74–0.85), with an overall N staging accuracy of 72% (95% CI: 0.67–0.77), and AUC of 0.797. Heterogeneity was substantial (I2 = 68.6–97.3%), but no publication bias was suggested.

**Conclusions:**

EUS provides robust diagnostic performance for T staging in gastric cancer, accurately assessing tumor invasion depth and reliably distinguishing early from advanced disease. However, moderate rates of overstaging and understaging persist, and nodal staging performance remains suboptimal. These findings underscore the importance of operator experience, standardized staging protocols, and the potential value of adjunctive techniques, such as EUS-guided fine-needle aspiration or contrast-enhanced EUS to enhance diagnostic yield.

**Funding Agencies:**

None